# Regulation of Staphylococcal Enterotoxin-Induced Inflammation in Spleen Cells from Diabetic Mice by Polyphenols

**DOI:** 10.3390/microorganisms11041039

**Published:** 2023-04-15

**Authors:** Yuko Shimamura, Rina Noaki, Yukino Oura, Kenya Ichikawa, Toshiyuki Kan, Shuichi Masuda

**Affiliations:** 1 School of Food and Nutritional Sciences, University of Shizuoka, 52-1 Yada, Suruga-ku, Shizuoka 422-8526, Japan; shimamura@u-shizuoka-ken.ac.jp (Y.S.);; 2 Department of Synthetic Organic & Medicinal Chemistry, School of Pharmaceutical Sciences, University of Shizuoka, 52-1 Yada, Suruga-ku, Shizuoka 422-8526, Japan

**Keywords:** *Staphylococcus aureus*, staphylococcal enterotoxin A, diabetes, inflammation, diabetes, polyphenols

## Abstract

Patients with diabetes are known to be more susceptible to infections following the establishment of *Staphylococcus aureus* in their nasal passages and on their skin. The present study evaluated the effects of staphylococcal enterotoxin A (SEA) on the immune responses of spleen cells derived from diabetic mice, and examined the effects of polyphenols, catechins, and nobiletin on inflammation-related gene expression associated with the immune response. (−)-Epigallocatechin gallate (EGCG), possessing hydroxyl groups, interacted with SEA, whereas nobiletin, possessing methyl groups, did not interact with SEA. The exposure of spleen cells derived from diabetic mice to SEA enhanced the expression of interferon gamma, suppressor of cytokine signaling 1, signal transducer and activator of transcription 3, interferon-induced transmembrane protein 3, Janus kinase 2, and interferon regulatory factor 3, suggesting that SEA sensitivity is variable in the development of diabetes. Both EGCG and nobiletin changed the expression of genes related to SEA-induced inflammation in spleen cells, suggesting that they inhibit inflammation through different mechanisms. These results may lead to a better understanding of the SEA-induced inflammatory response during diabetogenesis, and the establishment of methods to control these effects with polyphenols.

## 1. Introduction

*Staphylococcus aureus* is an important human pathogen that causes toxin-type food poisoning and a variety of infectious symptoms, including life-threatening conditions. *S. aureus* is known to be pathogenic to humans and animals, causing pyogenic skin disease and toxic shock syndrome [1]. *S. aureus* produces more than 20 types of staphylococcal enterotoxins (SEs); among these, SEA has been reported to have emetic and superantigen activity [2]. When SEA invades the body, cytokines are produced by phagocytic cells such as macrophages and neutrophils, followed by the induction of acute inflammation [3]. SEA cross-links major histocompatibility complex (MHC) class II molecules expressed by antigen-presenting cells with T-cell receptors, resulting in T-cell proliferation and toxic shock syndrome [4,5,6]. It has also been suggested that SEA binds to the cytokine receptor gp130 and activates signal transducer and activator of transcription 3 (STAT3) [7], which induces chronic inflammation and potentially causes insulin resistance [8]. Superantigen toxins produced by *S. aureus* have also been reported to induce insulin resistance, impaired glucose tolerance, and systemic inflammation in rabbits [7]. Furthermore, patients with diabetes are known to be more susceptible to infections following the establishment of *S. aureus* in their nasal passages and on their skin [9,10]. It has been reported that *S. aureus* activates a virulence-expressing system in response to environmental changes occurring in hosts with diabetes, differing from the response to infection in normal hosts [11]. However, the effect of *S. aureus* virulence factors on the immune response of patients with diabetes has not been clarified in detail.

In adipocytes and hepatocytes, IL-6 binds to gp130 and induces the expression of suppressor of cytokine signaling 1 (SOCS1) via the Janus kinase (JAK)/STAT pathway. SOCS1 inhibits insulin action by blocking tyrosine phosphorylation of insulin receptor substrate 1 [12]. Therefore, SEA may contribute to the induction of insulin resistance and diabetes by inducing STAT3 phosphorylation. In addition, many inflammation-related diseases, such as Crohn’s disease, pleurisy, and psoriasis, have been attributed to excessive activation of the JAK/STAT pathway [13,14,15]. Furthermore, the JAK/STAT pathway plays an important role in the regulation of critical functions such as cell proliferation, differentiation, survival, and apoptosis; immune responses; and homeostasis. Therefore, control of the overexpression or activation of JAK/STAT signaling is an emerging strategy for treating the aforementioned diseases. In a previous study, SEA was found to induce Th1 cytokines such as interferon gamma (IFN-γ) [16], suggesting that SEA plays an important role in the induction of inflammation in patients with diabetes. IFN-induced transmembrane protein 3 (IFITM3) is stimulated by type II (γ) interferons (IFNs), and upregulation of the IFITM3 gene is induced via the transcription factor IRF3 [17]. However, the mechanism of action of SEA on the JAK/STAT pathway, an intracellular signaling pathway for IFN, and the associated expression system of IFN are unknown. Elucidating the mechanisms by which *S. aureus* induces chronic inflammation in patients with diabetes could contribute to the establishment of methods to control diabetes-related diseases.

Previous studies illustrated that the hydroxyl group at position 3″ of the galloyl group of (−)-epigallocatechin gallate (EGCG), a polyphenol found in green tea, interacts with Y91 of SEA [18]. It was also demonstrated that SEA enhances the level of STAT3 phosphorylation at Tyr705, and that the hydroxyl group at position 3″ of the galloyl group of EGCG is involved in the inhibition of this phosphorylation [19]. It has been reported that catechins inhibit the toxic activity of SEA by interacting with hydroxyl groups. In addition, nobiletin (hexamethoxyflavone), a flavone with highly methylated hydroxyl groups, also exhibits anti-inflammatory effects [20]. Nobiletin is contained in citrus peels, and it has been reported to exert diabetes-improving effects such as reducing obesity and reversing insulin resistance [21]. It was recently suggested that people with a higher polyphenol intake are less likely to develop diabetes [22], suggesting that polyphenols can inhibit the development of SEA-induced insulin resistance and chronic inflammation. However, there are no reports comparing and analyzing the interactions of SEA-targeted proteins with catechins and hexamethoxyflavones. Clarification of the interactions among the target molecules, SEA, and polyphenols is expected to provide important information for elucidating their mechanisms of action, conducting structure–activity relationship studies, and improving selectivity to identify strategies for controlling SEA-induced inflammation.

**Figure 1:**
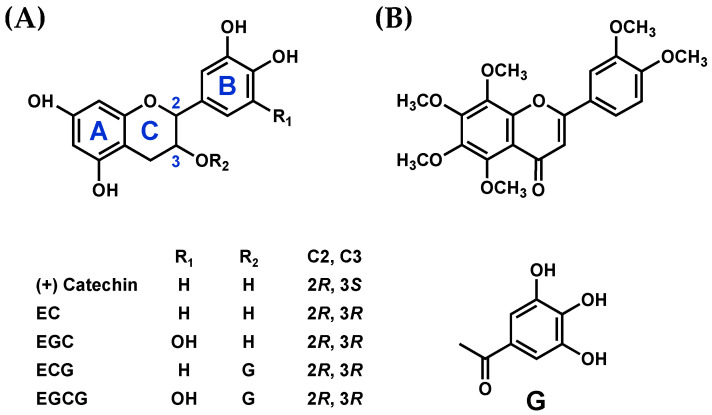
Polyphenols used in this study. (**A**) Structure of catechin. (**B**) Structure of nobiletin. G: galloyl group.

In this study, we analyzed the interaction of SEA with five catechins: ((+)-catechin, (−)-epicatechin [EC], (−)-epicatechin gallate [ECG], (−)-epigallocatechin [EGC], and EGCG) and nobiletin (Figure 1). Furthermore, the effects of SEA on the immune response of spleen cells from diabetic model mice, and the inhibitory effects of catechins and nobiletin on SEA-induced inflammation were analyzed.

## 2. Materials and Methods

### 2.1. Chemicals

SEA (95% pure; Toxin Technology Inc., Sarasota, FL, USA) was diluted using phosphate-buffered saline (PBS [pH 7.4], Thermo Fisher Scientific, Waltham, MA, USA). Streptozotocin (FUJIFILM Wako Pure Chemical Corporation, Osaka, Japan) solution (10 mg/mL) was prepared with 50 mmol/L citrate buffer (pH 4.5). Catechins [(+)-catechin, EC, ECG, EGC, and EGCG; all from Nagara Science Co., Ltd., Gifu, Japan] were diluted in dimethyl sulfoxide (DMSO; FUJIFILM Wako Pure Chemical Corporation). Nobiletin was synthesized in accordance with previous studies [23] and diluted in DMSO.

### 2.2. Assessment of the Interactions between SEA and Polyphenols Using the Thermal Shift Assay

Catechins and nobiletin were selected as ligands and diluted in DMSO. SEA (final concentration, 5 μg/mL) and each ligand (final concentration, 0.25 mM) were mixed and incubated at 37 °C for 2 h. Then, 18 µL of the mixture were mixed with 2 µL of SYPRO orange (dye, 60×) from the Protein Thermal Shift Dye Kit (Thermo Fisher Scientific, Baltics UAB, Vilnius, Lithuania). Samples were denatured by increasing the temperature from 25 °C to 99 °C at a rate of 1.6 °C/min using the StepOnePlus Real-Time PCR System (Applied Biosystems, Foster City, CA, USA). To determine *T*_m_ values, data were analyzed using Protein Thermal Shift Software v1.0 (Applied Biosystems). The software calculates *T*_m_ value from each fluorescence profile using the Boltzmann method (fluorescence intensity vs. temperature plot) and the derivative curve method [d(fluorescence)/dT vs. temperature plot (*T*_m_ D)]. In the differential curve method, *T*_m_ is calculated from the peak apex of the differential plot, and in the Boltzmann method, each value is calculated from the inflection point of the fluorescence plot. In this study, we analyzed the thermal denaturation temperature of SEA using the change in *T*_m_ (*T*_m_ D) of the differential curve method as an indicator.

### 2.3. Effect of pH on the Interactions between SEA and Polyphenols

McIlvaine buffer (Thermo Fisher Scientific) was adjusted to pH 4.0, 6.0, or 8.0 to examine the effect of pH. EGCG or nobiletin (final concentration, 3.0 mM) was added to SEA (final concentration, 5.0 µg/mL) diluted in McIlvaine buffer or PBS, and incubated at 37 °C for 24 h. Following centrifugation (4000× *g*, 5 min), the supernatant was collected. Milli-Q water was used as the control. Proteins were separated on acrylamide gels and transferred to a PVDF membrane, which was blocked with 5% skim milk powder (FUJIFILM Wako Pure Chemical Corporation). The PVDF membrane was soaked in rabbit anti-SEA (Sigma-Aldrich, St. Louis, MO, USA), diluted 1:1000 in PBS and incubated overnight at 4 °C. The PVDF membrane was washed with wash solution (20× Wash Solution Concentrate; KPL, Gaithersburg, MD, USA) three times for 5 min each with shaking at room temperature. The PVDF membrane was soaked in anti-mouse IgG (H + L) (KPL) diluted 1:10,000 in PBS at room temperature for 90 min with shaking. The membrane was washed with wash solution three times for 5 min each at room temperature with shaking. The PVDF membrane was then immersed in substrate solution (BCIP/NBT; KPL) and incubated at room temperature for 20 min to permit color development. The stained SEA bands were analyzed and quantified using ImageJ (National Institutes of Health, Bethesda, MA, USA).

### 2.4. Analysis of SEA-Induced Inflammation-Related Genes in Spleen Cells from Diabetic Mice

#### 2.4.1. Animals

Institute of Cancer Research (ICR) mice (5 weeks old, female) and C57BL/6J mice (5 weeks old, female) were purchased from Japan SLC Corporation (Hamamatsu, Japan) and reared in an animal center at 23 ± 1 °C with 55% ± 5% humidity under a 12-h/12-h light/dark cycle. The animals were granted ad libitum access to CE-2 (4.8% crude fat content; CLEA Japan, Inc., Tokyo, Japan) and tap water. The mice were acclimated for 1 week prior to use. The study was approved by the Institutional Animal Care and Use Committees of the University of Shizuoka (permit number: 20527, date of approval: 1 April 2020; permit number: 215320, date of approval: 30 March 2021).

#### 2.4.2. Preparation of the Diabetic Mouse Model

Streptozotocin solution was administered intraperitoneally to ICR mice at a dose of 200 mg/kg b.w. After 2 weeks, fasting blood glucose levels were measured, and animals with blood glucose levels exceeding 400 mg/dL were considered to have developed diabetes.

#### 2.4.3. Preparation of Mouse Spleen Cells

Mouse spleen cells were prepared from normal and diabetic mice as described in previous studies [16]. Spleen cells were cultured in RPMI 1640 medium containing 1% fetal bovine serum, 50 units/mL penicillin, 50 μg/mL streptomycin, 1 M sodium pyruvate, 20 mM l-glutamine, and 50 mM 2-mercaptoethanol (all from Thermo Fisher Scientific).

#### 2.4.4. SEA Exposure in Mouse Spleen Cells

Spleen cell solution containing 2.5 × 10^6^ cells/mL was added to cell culture medium (total volume, 4 mL) in six-well plates. For SEA exposure, 100 μL of SEA were added [final concentration, 50 and 100 ng/mL; (+) SEA]. Meanwhile, 100 μL of distilled water were used as a control [(−) SEA]. Cells were incubated for 16 h at 37 °C in a 5% CO_2_ atmosphere.

#### 2.4.5. Inhibitory Effects of Polyphenols on SEA-Induced Inflammation-Related Genes

Spleen cell solution containing 2.5 × 10^6^ cells/mL was added to cell culture medium (total volume, 4 mL) in six-well plates. Spleen cell solution was incubated with SEA (final concentration, 50 and 100 ng/mL), ECGG, or nobiletin (final concentration 50 μM) for 16 h at 37 °C in a 5% CO_2_ atmosphere.

#### 2.4.6. Total RNA Extraction and Real-Time RT-PCR

Total RNA was extracted using an RNeasy mini Kit (QIAGEN, Hilden, Germany). Total RNA was converted into cDNA using a PrimeScript RT Reagent Kit (Takara, Kusatsu, Japan). To examine the relative mRNA expression of genes after SEA exposure, each prepared cDNA was analyzed by real-time RT-PCR performed on a Real-time PCR instrument (Thermal Cycler Dice^®^ Real-Time System II; Takara), using the SYBER ExScript RT-PCR kit (Takara) according to the manufacturer’s instructions. The hypoxanthine–guanine phosphoribosyltransferase (Hprt) gene was used as an internal standard to normalize mRNA levels between samples. The primer sequences used are shown in Table 1. Heatmaps were prepared using Heatmapper (http://www.heatmapper.ca/ (accessed on 24 February 2023)) [24]. The changes in gene expression were shown as the average of the results using splenocytes prepared from the spleens of at least three different mice.

### 2.5. Statistical Analysis

Data were analyzed by one-way analysis of variance followed by Dunnett’s test using Microsoft Excel 2019 (Microsoft, Redmond, WA, USA). The significance level was set at *p* < 0.05, and all experiments were repeated at least three times.

## 3. Results

### 3.1. Interaction Analysis of SEA and Polyphenols

**Figure 2:**
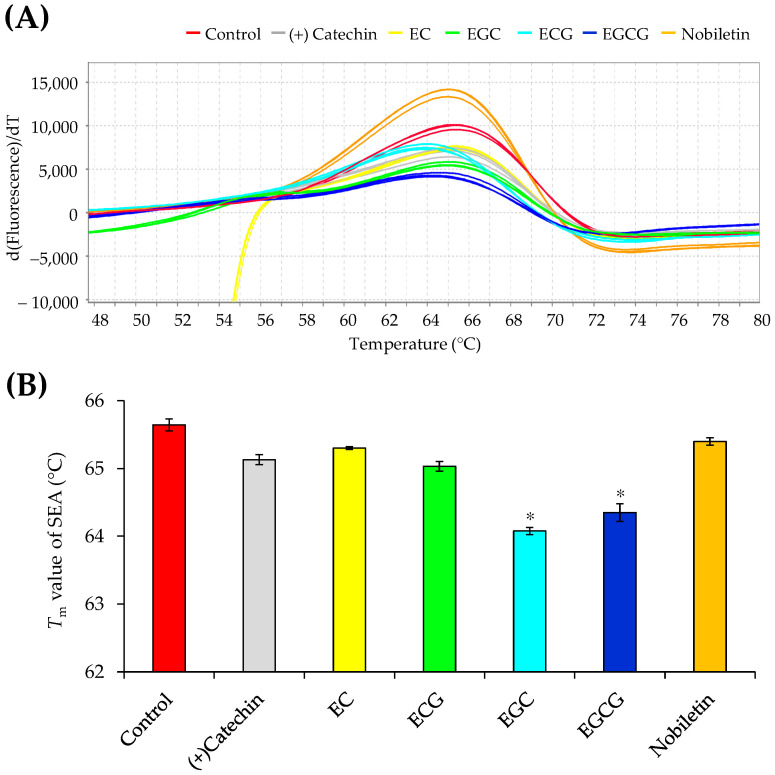
Interactions between SEA and polyphenols. (**A**) Melting profile of SEA in the presence of polyphenols. The images show the profile of the derivative of fluorescence emission as a function of temperature [d(Fluorescence)/dT]. SEA was used as the protein, and each polyphenol served as the ligand. The compounds were reacted at 37 °C for 2 h before the interaction was analyzed using the protein thermal shift assay. (**B**) Melting temperature of SEA in the presence of polyphenols. * *p* < 0.05 compared to the control. The data are presented as the mean ± standard deviation of four independent experiments.

The interactions of SEA with six polyphenols, (+)-catechin, EC, ECG, EGC, EGCG, and nobiletin, were analyzed by the protein thermal shift assay. Compared to that of SEA alone (65.36 °C), *T*_m_ of SEA was unchanged in the presence of (+)-catechin, EC, ECG, and nobiletin. Conversely, *T*_m_ of SEA was significantly different in the presence of EGC (63.89 °C) and EGCG (64.36 °C). Thus, among the polyphenols tested, only EGC and EGCG interacted with SEA (Figure 2).

### 3.2. Effect of pH on the Interaction between SEA and Polyphenols

**Figure 3:**
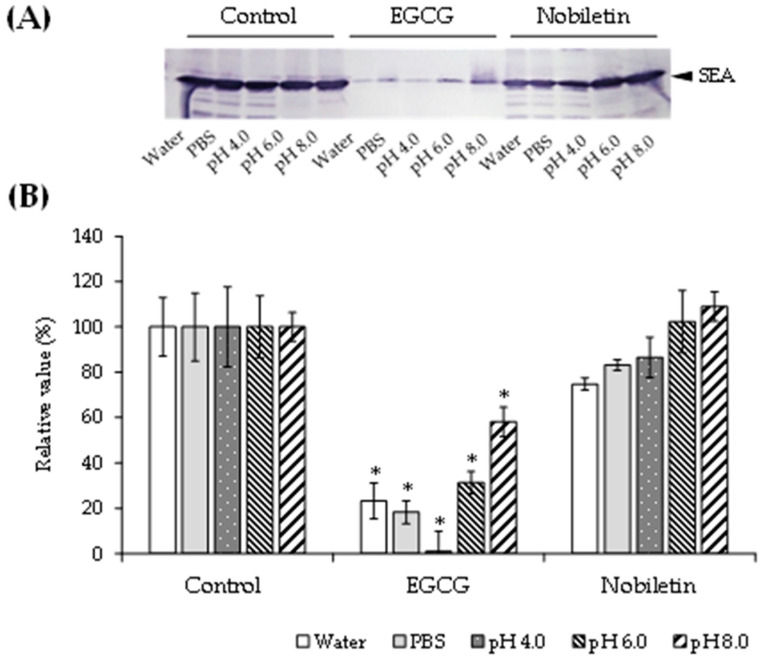
The effect of pH on the interaction between SEA and polyphenols. All samples were prepared at a final concentration of 3.0 mM. Each test sample was mixed with SEA (5.0 μg/mL) in 100 μL of Milli-Q water, PBS (pH 7.2), or McIlvaine buffer (pH 4.0, 6.0, or 8.0) and incubated at 37 °C for 24 h. Following centrifugation (4000× *g*, 5 min), the supernatant was applied to SDS-PAGE and visualized by Western blotting. Milli-Q water was used as a positive control. * *p* < 0.05 vs. the control. The values represent the mean ± standard deviation of three independent experiments.

After reacting SEA with EGCG or nobiletin in pH 4.0, 6.0, or 8.0 buffer for 2 h, the interaction was examined by Western blotting. The results illustrated that the interaction of SEA with EGCG was weaker at pH 6 and 8 than that in PBS (Figure 3). By contrast, nobiletin, which did not interact with SEA in PBS, did not change the SEA band at any pH (Figure 3).

### 3.3. SEA-Induced Inflammation in Normal Mouse Spleen Cells

**Figure 4:**
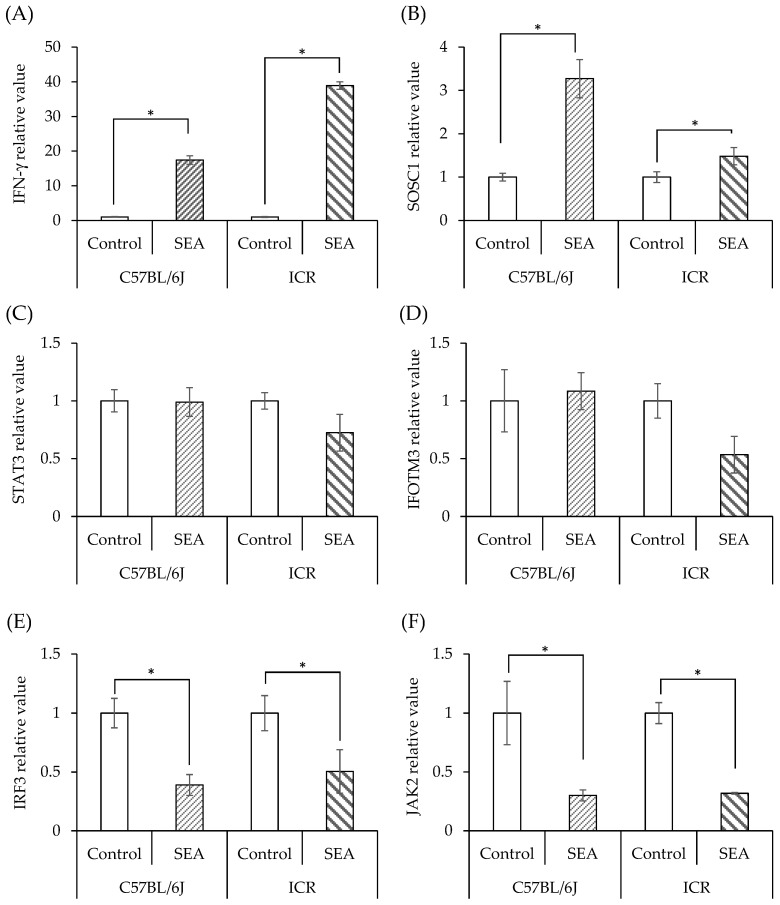
Comparison of SEA-induced JAK/STAT pathway-related gene expression in spleen cells from normal C57BL/6J and ICR mice. (**A**) IFN-γ, (**B**) SOCS1, (**C**) STAT3, (**D**) IFITM3, (**E**) IRF3, (**F**) JAK2. Spleen cells from normal mice (C57BL/6J and ICR mice) were exposed to SEA (50 ng/mL), and the expression of JAK/STAT pathway-related genes was examined by real-time RT-PCR. Gene expression was normalized to Hprt gene expression. The fold change was determined relative to the control without SEA. * *p* < 0.05 compared to the control. The values represent the mean ± standard deviation of three independent experiments.

Spleen cells from two types of normal mice (C57BL/6J and ICR mice) were exposed to SEA, and the expression of JAK/STAT pathway-related genes induced by SEA was examined. IFN-γ (Figure 4A) and SOCS1 (Figure 4B) expression was increased by SEA exposure in both cell lines. STAT3 expression was not changed by SEA exposure in either cell line (Figure 4C). IFITM3 expression was decreased by SEA exposure only in ICR mouse spleen cells, albeit without significance (Figure 4D). IRF3 and JAK2 expression was reduced by SEA exposure in both cell lines (Figure 4E,F). From these results, there was little difference in the ability of SEA to induce inflammation in spleen cells derived from two mouse strains. Therefore, diabetic mice were prepared using only ICR mice, and the changes in inflammation-related genes by SEA in spleen cells under diabetic conditions were evaluated.

### 3.4. SEA-Induced Inflammation in Spleen Cells from Mice with Diabetes

**Figure 5:**
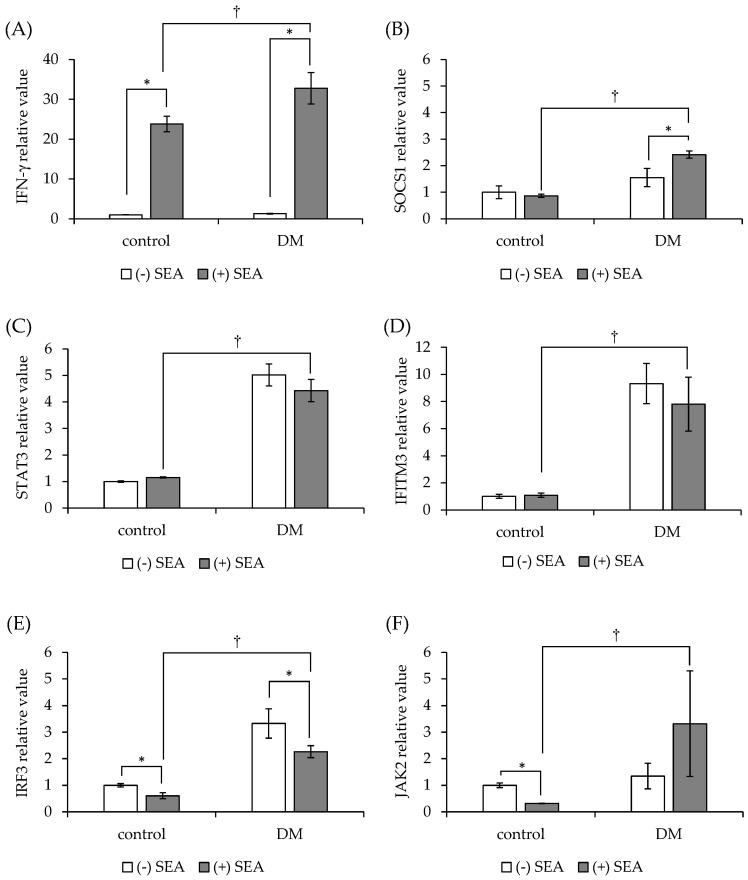
Comparison of SEA-induced JAK/STAT pathway-related gene expression in spleen cells from normal mice and diabetic mice (DM). (**A**) IFN-γ, (**B**) SOCS1, (**C**) STAT3, (**D**) IFITM3, (**E**) IRF3, (**F**) JAK2. Spleen cells from normal mice (control) and DM were exposed to SEA (50 ng/mL), and the expression of JAK/STAT pathway-related genes was examined by real-time RT-PCR. Gene expression was normalized to that of the Hprt gene. The fold change was determined relative to the control without SEA. * *p* < 0.05 compared to (−) SEA, † *p* < 0.05 compared to the control. The values represent the mean ± standard deviation of three independent experiments.

Spleen cells from diabetic mice were exposed to SEA, and the expression of JAK/STAT pathway-related genes was examined. Compared to the findings in normal mice, the expression of all genes was significantly increased in diabetic mouse-derived spleen cells by exposure to SEA. The expression of IFN-γ (Figure 5A) and SOCS1 (Figure 5B) was significantly increased in diabetic mouse-derived spleen cells following SEA exposure in comparison to that in control cells. STAT3, IFITM3, and JAK2 expression in diabetic mouse-derived spleen cells was not changed by SEA exposure (Figure 5C,D,F). SEA treatment was linked to a significant decrease in IRF3 expression (Figure 5E). The expression patterns of SOCS1 and JAK2 following SEA exposure in spleen cells differed between normal and diabetic mice.

### 3.5. Inhibitory Effects of Polyphenols on SEA-Induced Inflammation-Related Genes in the Spleen Cells of Diabetic Mice

**Figure 6:**
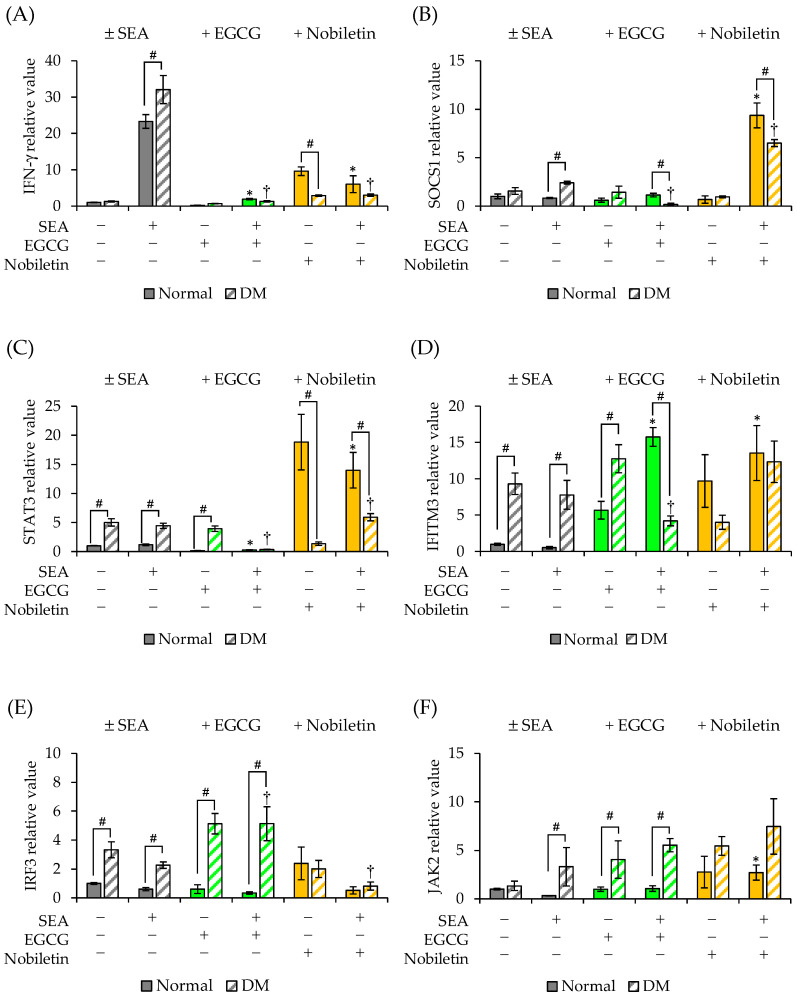
The effect of polyphenols on the SEA-induced expression of JAK/STAT pathway-related genes in spleen cells from normal mice and diabetic mice (DM). (**A**) IFN-γ, (**B**) SOCS1, (**C**) STAT3, (**D**) IFITM3, (**E**) IRF3, (**F**) JAK2. Spleen cells from normal mice (control) and DM were exposed to different combinations of SEA (50 ng/mL), EGCG (0.05 mM), and nobiletin (0.05 mM), and the expression of JAK/STAT pathway-related genes was examined by real-time RT-PCR. Gene expression was normalized to that of the Hprt gene. The fold change was determined relative to the control without SEA. * *p* < 0.05 compared to normal—SEA, † *p* < 0.05 compared to DM—SEA, # *p* < 0.05 compared to normal. The values represent the mean ± standard deviation of three independent experiments.

**Figure 7:**
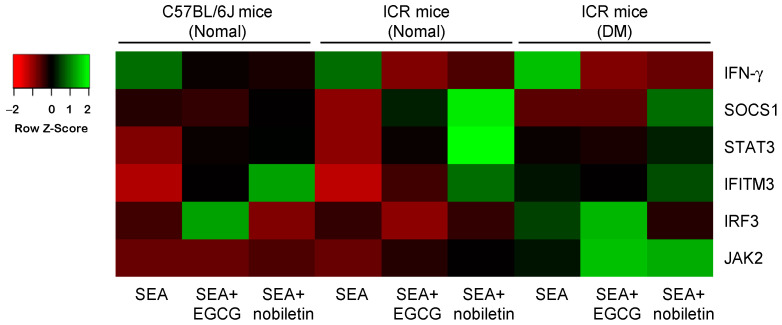
The effect of EGCG and nobiletin on SEA-induced changes in JAK/STAT pathway-related gene expression. Spleen cells from normal mice and diabetic mice (DM) were exposed to SEA, EGCG, and nobiletin. The results for C57BL/6J mice (normal) are shown only for the group treated with EGCG. The fold change was determined relative to the control without SEA treatment. Differential expression of mRNA is shown by the intensity of green (upregulation) versus red (downregulation).

EGCG and nobiletin significantly inhibited SEA-induced IFN-γ gene expression in both normal mouse- and diabetic mouse-derived spleen cells (Figure 6A and Figure 7). EGCG inhibited SEA-induced SOCS1 expression only in spleen cells derived from DM (Figure 6B and Figure 7). By contrast, nobiletin further increased SEA-induced SOCS1 expression in both normal mouse- and diabetic mouse-derived spleen cells. EGCG decreased STAT3 expression only in the presence of SEA in both spleen cell types (Figure 6C and Figure 7). Conversely, nobiletin significantly increased STAT3 expression in both spleen cell types irrespective of SEA. EGCG inhibited SEA-induced IFITM3 expression only in spleen cells from DM (Figure 6D and Figure 7). However, nobiletin increased SEA-induced IFITM3 expression in both spleen cell types. EGCG upregulated IRF3 expression only in diabetic mouse-derived spleen cells irrespective of SEA (Figure 6E and Figure 7). Nobiletin reduced SEA-induced IRF3 expression only in spleen cells derived from DM. EGCG did not change SEA-induced JAK2 gene expression in either spleen cell type (Figure 6F and Figure 7). Nobiletin inhibited the SEA-induced decrease in JAK2 expression normal mouse-derived spleen cells.

## 4. Discussion

EGCG has been demonstrated to bind to proteins in saliva [25] and many different proteins in cells [26]. Various polyphenols other than EGCG have been reported to bind to and aggregate with proteins such as casein in foods [27]. Plant-derived components binding to SEA were identified in prior studies, and several polyphenols have been found to inhibit SEA production or its toxin activity [28,29]. Evaluating the interaction of polyphenols with the target molecule of SEA [30] is expected to provide important information for elucidating its mechanism of action, structure–activity relationships, and selectivity, as well as for devising strategies to control SEA-induced inflammation. In this study, the interactions of SEA proteins with catechins and nobiletin were analyzed. The results revealed that EGC and EGCG, but not nobiletin, interacted with SEA. In a previous study, SEA was found to interact with the hydroxyl group at position 3″ of the galloyl group of EGCG [12]. It was suggested that nobiletin might not be able to interact with SEA because all of its hydroxyl groups are methylated to methoxy groups. Catechins are also known to be stable in acidic conditions, but to oxidatively decompose in neutral to slightly alkaline conditions [31]. Cis-type catechins were reported to be most stable at pH 4.0–5.2, with accelerated degradation occurring at pH ≥ 5.2 [32]. The interaction between SEA and EGCG was weaker at pH 8.0, suggesting that the degradation of EGCG influenced its interaction with SEA.

Therefore, the difference in the expression of SEA-inducible genes in diabetic mouse-derived spleen cells between EGCG, which interacts with SEA, and nobiletin, which does not interact with SEA, was examined. In a previous study, the toxin activity of SEA was evaluated using spleen cells obtained from C57BL/6 mice, an inbred strain. Therefore, initially, SEA-induced inflammatory responses were examined using spleen cells derived from C57BL/6 and ICR mice. The results showed no strain differences in SEA sensitivity. In addition, it has been reported that there are strain differences in the induction of diabetes by STZ in mice. Among ddY, BALB/c, and C57BL/6 mice, elevated serum glucose levels at 12 weeks after STZ administration have been observed only in ddY and ICR mice [33]. Therefore, in this study, to evaluate the induction of inflammation in splenocytes, type 1 diabetes model mice were created not from C57BL/6J mice but from ICR mice, which have few individual differences and stably induce a hyperglycemic state.

Compared to the findings in normal mice, FN-γ, SOCS1, STAT3, IFITM3, IRF3, and JAK2 expression was significantly higher in spleen cells from mice with diabetes, suggesting homeostatic inflammation was induced. It has been reported that IFN-γ upregulation and JAK/STAT1 pathway activation contribute to adipocyte dysfunction and insulin resistance [34]. In addition, IRF3 is an important transcriptional effector of the inflammatory response associated with insulin resistance and nutrient overload [35], and the expression of these genes was similarly observed in the present study. Thus, the expression of genes related to the JAK/STAT pathway in mouse spleen cells is upregulated during the development of diabetes mellitus.

SEA-treated spleen cells from mice with diabetes exhibited little change in the expression of STAT3, IFITM3, and JAK2. However, the expression of IFN-γ and SOCS1 increased, and that of IRF3 decreased. In normal mouse-derived spleen cells, SEA exposure did not change the expression of SOCS1, whereas its expression was increased in diabetic mouse-derived spleen cells. The development of type 2 diabetes is caused by the overexpression of SOCS proteins [36] and activation of the nuclear factor kappa B, mitogen-activated protein kinase, and JAK/STAT pathways, which promote local cytokine and chemokine production and release, resulting in progressive inflammation [34,37]. IFN-γ has been reported to induce insulin resistance by activating STAT1 in adipocytes, decreasing the phosphorylation of the serine/threonine kinase, and downregulating insulin receptor substrate 1 and glucose transporter type 4 [30]. STAT3 expression was not changed by SEA exposure in diabetic mouse-derived spleen cells. STAT3 is activated primarily by IL-6 and other gp130-related cytokines [38]. A previous study found that SEA promotes STAT3 phosphorylation [19], and our results suggest that SEA promotes STAT3 phosphorylation without suppressing its mRNA expression. SEA increased the expression of genes involved in the pathogenesis of diabetes, such as IFN-γ and SOCS1, suggesting that SEA plays an important role in inducing inflammation in diabetic mouse-derived spleen cells.

The effects of EGCG and nobiletin on JAK/STAT pathway-related genes induced by SEA were studied using diabetic mouse-derived spleen cells. EGCG and nobiletin significantly decreased SEA-induced IFN-γ expression in diabetic mouse-derived spleen cells. Nobiletin more strongly inhibited SEA-induced IFN-γ expression in diabetic mouse-derived spleen cells than in normal mouse-derived spleen cells. Nobiletin has been reported to improve hyperglycemia and insulin resistance [21], supporting these results. Exposure of diabetic mouse-derived spleen cells to SEA resulted in little change in SOCS1 and STAT3 expression, whereas upon the addition of EGCG, their expression was comparable to that in spleen cells from normal mice. Conversely, nobiletin further increased SEA-induced SOCS1 and STAT3 expression in spleen cells from normal and DM. SOCS1 inhibits JAK phosphorylation and its function, and its promoter region contains a putative STAT3 binding site [39,40]. In animal models of type 1 diabetes, SOCS1 has been suggested to be involved in the regulation of inflammatory cytokines such as IFN-γ and tumor necrosis factor alpha, which are responsible for the destruction of pancreatic β cells, and SOCS1 may protect β cells from cytokine-mediated destruction [41,42]. Thus, nobiletin may contribute to the regulation of T-cell inflammation mediated by SOCS1.

The effects of EGCG and nobiletin on SEA-induced IFITM3 and IRF3 expression in spleen cells from diabetic mice were different. EGCG inhibited SEA-induced IFITM3 expression in spleen cells from diabetic mice, whereas nobiletin suppressed SEA-induced IRF3 expression. IRF3 induces the transcription of type 1 IFNs after their phosphorylation, dimerization, and translocation to the nucleus. Jiang et al. reported that the autophagosome-dependent degradation of IRF3 via IFITM3 induces an inverse correlation between IRF3 and IFITM3 levels [17]. It was suggested that EGCG can suppress IFITM3 expression, followed by the inhibition of IRF3 expression. EGCG and nobiletin did not change JAK2 gene expression in spleen cells from diabetic mice. These results suggest that EGCG and nobiletin have different inhibitory effects on inflammation induced by diabetogenesis. The present study revealed a partial mechanism of SEA-induced activation of the JAK/STAT pathway under diabetic conditions, and its suppression by EGCG and nobiletin. In the future, the mechanisms of the inhibitory effects of EGCG and nobiletin on the induction of inflammation-related genes by SEA should be further elucidated. Further research is expected to establish new strategies for preventing various diseases caused by SEA using polyphenols.

## Figures and Tables

**Table 1:** Primers for real-time RT-PCR were used in this study.

Primer	Forward Primer (5^′^–3^′^)	Reverse Primer (5^′^–3^′^)
Hprt	GTTGGATACAGGCCAGACTTTGTTG	GAGGGTAGGCTGGCCTATAGGCT
IFN-γ	CATTGAAAGCCTAGAAAGTCTG	CTCATGAATGCATCCTTTTTCG
SOCS1	GATTCTGCGTGCCGCTCT	TGCGTGCTACCATCCTACTC
STAT3	CTACCTCTACCCCGACATTCC	GATGAACTTGGTCTTCAGGTACG
JAK2	GGAATGGCCTGCCTTACAATG	TGGCTCTATCTGCTTCACAGAAT
IFITM3	GAGTGGCTGTAGCACCAACA	GCGGAGCAAAGGCAGCAC
IRF3	CGGAAAGAAGTGTTGCGGTT	TTTTCCTGGGAGTGAGGCAG

Hprt was used as an internal standard.

## Data Availability

The data presented in this work are available in this article.

## References

[1] Omoe K., Hu D.L., Takahashi-Omoe H., Nakane A., Shinagawa K. (2003). Identification and characterization of a new staphylococcal enterotoxin-related putative toxin encoded by two kinds of plasmids. Infect. Immun..

[2] Ono H.K., Hirose S., Narita K., Sugiyama M., Asano K., Hu D.L., Nakane A. (2019). Histamine release from intestinal mast cells induced by staphylococcal enterotoxin a (SEA) evokes vomiting reflex in common marmoset. PLoS Pathog..

[3] Liu X., Wen Y., Zhao Z., Jeffry J., Zeng L., Zou Z., Chen H., Tao A. (2020). Synergistic activation of Src, ERK and STAT pathways in PBMCs for Staphylococcal enterotoxin A induced production of cytokines and chemokines. Asian Pacific J. Allergy Immunol..

[4] Heinrich P.C., Behrmann I., Haan S., Hermanns H.M., Müller-Newen G., Schaper F. (2003). Principles of interleukin (IL)-6-type cytokine signalling and its regulation. Biochem. J..

[5] Lagathu C., Bastard J.P., Auclair M., Maachi M., Capeau J., Caron M. (2003). Chronic interleukin-6 (IL-6) treatment increased IL-6 secretion and induced insulin resistance in adipocyte: Prevention by rosiglitazone. Biochem. Biophys. Res. Commun..

[6] Banke E., Rödström K., Ekelund M., Dalla-Riva J., Lagerstedt J.O., Nilsson S., Degerman E., Lindkvist-Petersson K., Nilson B. (2014). Superantigen activates the gp130 receptor on adipocytes resulting in altered adipocyte metabolism. Metabolism.

[7] Vu B.G., Stach C.S., Kulhankova K., Salgado-Pabón W., Klingelhutz A.J., Schlievert P.M. (2015). Chronic superantigen exposure induces systemic inflammation, elevated bloodstream endotoxin, and abnormal glucose tolerance in rabbits: Possible role in diabetes. MBio.

[8] Smit J., Søgaard M., Schønheyder H.C., Nielsen H., Frøslev T., Thomsen R.W. (2016). Diabetes and risk of community-acquired *Staphylococcus aureus* bacteremia: A population-based case-control study. Eur. J. Endocrinol..

[9] Joshi N., Caputo G.M., Weitekamp M.R., Karchmer A.W. (1999). Infections in patients with diabetes mellitus. N. Engl. J. Med..

[10] Dunyach-Remy C., Ngba Essebe C., Sotto A., Lavigne J.P. (2016). *Staphylococcus aureus* toxins and diabetic foot ulcers: Role in pathogenesis and interest in diagnosis. Toxins.

[11] Miyazaki S., Matsumoto Y., Sekimizu K., Kaito C. (2012). Evaluation of *Staphylococcus aureus* virulence factors using a silkworm model. FEMS Microbiol. Lett..

[12] Liau N.P.D., Laktyushin A., Lucet I.S., Murphy J.M., Yao S., Whitlock E., Callaghan K., Nicola N.A., Kershaw N.J., Babon J.J. (2018). The molecular basis of JAK/STAT inhibition by SOCS1. Nat. Commun..

[13] Danese S., Mantovani A. (2010). Inflammatory bowel disease and intestinal cancer: A paradigm of the Yin-Yang interplay between inflammation and cancer. Oncogene.

[14] Atreya R., Neurath M.F. (2008). Signaling molecules: The pathogenic role of the IL-6/STAT-3 trans signaling pathway in intestinal inflammation and in colonic cancer. Curr. Drug Targets.

[15] Mariotto S., Esposito E., di Paola R., Ciampa A., Mazzon E., de Prati A.C., Darra E., Vincenzi S., Cucinotta G., Caminiti R. (2008). Protective effect of Arbutus unedo aqueous extract in carrageenan-induced lung inflammation in mice. Pharmacol. Res..

[16] Shimamura Y., Utsumi M., Hirai C., Kurokawa A., Kan T., Ohashi N., Masuda S. (2020). Effect of (−)-epigallocatechin gallate to staphylococcal enterotoxin A on toxin activity. Molecules.

[17] Jiang L.Q., Xia T., Hu Y.H., Sun M.S., Yan S., Lei C.Q., Shu H.B., Guo J.H., Liu Y. (2018). IFITM3 inhibits virus-triggered induction of type I interferon by mediating autophagosome-dependent degradation of IRF3. Cell. Mol. Immunol..

[18] Shimamura Y., Utsumi M., Hirai C., Nakano S., Ito S., Tsuji A., Ishii T., Hosoya T., Kan T., Ohashi N. (2018). Binding of catechins to staphylococcal enterotoxin A. Molecules.

[19] Shimamura Y., Noaki R., Kurokawa A., Utsumi M., Hirai C., Kan T., Masuda S. (2021). Effect of (−)-epigallocatechin gallate on activation of JAK/STAT signaling pathway by staphylococcal enterotoxin A. Toxins.

[20] Yoshigai E., Machida T., Okuyama T., Mori M., Murase H., Yamanishi R., Okumura T., Ikeya Y., Nishino H., Nishizawa M. (2013). Citrus nobiletin suppresses inducible nitric oxide synthase gene expression in interleukin-1β-treated hepatocytes. Biochem. Biophys. Res. Commun..

[21] Lee Y.S., Cha B.Y., Saito K., Yamakawa H., Choi S.S., Yamaguchi K., Yonezawa T., Teruya T., Nagai K., Woo J.T. (2010). Nobiletin improves hyperglycemia and insulin resistance in obese diabetic ob/ob mice. Biochem. Pharmacol..

[22] PREDIMED Study Investigators (2015). Intake of total polyphenols and some classes of polyphenols is inversely associated with diabetes in elderly people at high cardiovascular disease risk. J. Nutr..

[23] Asakawa T., Hiza A., Nakayama M., Inai M., Oyama D., Koide H., Shimizu K., Wakimoto T., Harada N., Tsukada H. (2011). PET imaging of nobiletin based on a practical total synthesis. Chem. Commun..

[24] Babicki S., Arndt D., Marcu A., Liang Y., Grant J.R., Maciejewski A., Wishart D.S. (2016). Heatmapper: Web-enabled heat mapping for all. Nucleic Acids Res..

[25] Canon F., Paté F., Cheynier V., Sarni-Manchado P., Giuliani A., Pérez J., Durand D., Li J., Cabane B. (2013). Aggregation of the salivary proline-rich protein IB5 in the presence of the tannin EgCG. Langmuir.

[26] Garbisa S., Biggin S., Cavallarin N., Sartor L., Benelli R., Albini A. (1999). Tumor invasion: Molecular shears blunted by green tea. Nat. Med..

[27] Bourassa P., Bariyanga J., Tajmir-Riahi H.A. (2013). Binding sites of resveratrol, genistein, and curcumin with milk α-and β-caseins. J. Phys. Chem..

[28] Shimamura Y., Aoki N., Sugiyama Y., Tanaka T., Murata M., Masuda S. (2016). Plant-derived polyphenols interact with staphylococcal enterotoxin A and inhibit toxin activity. PLoS ONE.

[29] Shimamura Y., Hirai C., Sugiyama Y., Utsumi M., Yanagida A., Murata M., Ohashi N., Masuda S. (2017). Interaction between various apple procyanidin and staphylococcal enterotoxin A and their inhibitory effects on toxin activity. Toxins.

[30] McGillicuddy F.C., Chiquoine E.H., Hinkle C.C., Kim R.J., Shah R., Roche H.M., Smyth E.M., Reilly M.P. (2009). Interferon γ attenuates insulin signaling, lipid storage, and differentiation in human adipocytes via activation of the JAK/STAT pathway. J. Biol. Chem..

[31] Ho Y., Lee Y.L., Hsu K.Y. (1995). Determination of (+)-catechin in plasma by high-performance liquid chromatography using fluorescence detection. J. Chromatogr. B Biomed. Appl..

[32] Li N., Taylor L.S., Ferruzzi M.G., Mauer L.J. (2012). Kinetic study of catechin stability: Effects of Ph, concentration, and temperature. J. Agric. Food Chem..

[33] Hayashi K., Kojima R., Ito M. (2006). Strain differences in the diabetogenic activity of streptozotocin in mice. Biol. Pharm. Bull..

[34] Esser N., Legrand-Poels S., Piette J., Scheen A.J., Paquot N. (2014). Inflammation as a link between obesity, metabolic syndrome and type 2 diabetes. Diabetes Res. Clin. Pract..

[35] Kumari M., Wang X., Lantier L., Lyubetskaya A., Eguchi J., Kang S., Tenen D., Roh H.C., Kong X., Kazak L. (2016). IRF3 promotes adipose inflammation and insulin resistance and represses browning. J. Clin. Investig..

[36] Zimeri J., Tong C.H. (1999). Degradation kinetics of (−)-epigallocatechin gallate as a function of pH and dissolved oxygen in a liquid model system. J. Food Sci..

[37] Turner M.D. (2017). The identification of TNFR5 as a therapeutic target in diabetes. Expert Opin. Ther. Targets.

[38] Yoshimura A. (2006). Signal transduction of inflammatory cytokines and tumor development. Cancer Sci..

[39] Naka T., Narazaki M., Hirata M., Matsumoto T., Minamoto S., Aono A., Nishimoto N., Kajita T., Taga T., Yoshizaki K. (1997). Structure and function of a new STAT-induced STAT inhibitor. Nature.

[40] Saito H., Morita Y., Fujimoto M., Narazaki M., Naka T., Kishimoto T. (2000). IFN regulatory factor-1-mediated transcriptional activation of mouse STAT-induced STAT inhibitor-1 gene promoter by IFN-gamma. J. Immunol..

[41] Chong M.M., Chen Y., Darwiche R., Dudek N.L., Irawaty W., Santamaria P., Allison J., Kay T.W., Thomas H.E. (2004). Suppressor of cytokine signalling-1 overexpression protects pancreatic beta cells from CD8^+^ T cell-mediated autoimmune destruction. J. Immunol..

[42] Hultcrantz M., Jacobson S., Hill N.J., Santamaria P., Flodström-Tullberg M. (2003). Target cell expression of suppressor of cytokine signalling-1 prevents diabetes in the NOD mouse. Diabetes.

